# Fc engineering of a fully humanized anti-CD147 monoclonal antibody enhances ADCC against T-cell acute lymphoblastic leukemia and T-lymphoblastic lymphoma

**DOI:** 10.1038/s41598-026-52003-x

**Published:** 2026-05-08

**Authors:** Zaw Ye Htet, Thanathat Pamonsupornwichit, Kanokporn Sornsuwan, Natsima Viriyaadhammaa, Phatcharida Jantaree, Umpa Yasamut, Nutjeera Intasai, Chatchai Tayapiwatana

**Affiliations:** 1 Division of Clinical Microscopy, Department of Medical Technology, Faculty of Associated Medical Sciences, Chiang Mai University, Chiang Mai, 50200 Thailand; 2 Center of Biomolecular Therapy and Diagnostic, Faculty of Associated Medical Sciences, Chiang Mai University, Chiang Mai, 50200 Thailand; 3 Office of Research Administration, Chiang Mai University, Chiang Mai, 50200 Thailand; 4 Center of Multidisciplinary Technology for Advanced Medicine (CMUTEAM), Faculty of Medicine, Chiang Mai University, Chiang Mai, 50200 Thailand; 5 Department of Biochemistry, Faculty of Medicine, Chiang Mai University, Chiang Mai, 50200 Thailand; 6 Division of Clinical Immunology, Department of Medical Technology, Faculty of Associated Medical Sciences, Chiang Mai University, Chiang Mai, 50200 Thailand

**Keywords:** T-ALL, T-LBL, CD147, Fc engineering, Humanized antibody, ADCC, Cancer, Immunology, Oncology

## Abstract

T-cell acute lymphoblastic leukemia (T-ALL) and T-lymphoblastic lymphoma (T-LBL) are aggressive hematologic malignancies with limited targeted treatment options and poor clinical outcomes across all age groups. CD147 is a multifunctional transmembrane glycoprotein that is highly expressed in various cancers, including T-cell leukemia and lymphoma. Its involvement in tumor progression and immune evasion makes CD147 a promising target for cancer immunotherapy. Monoclonal antibody-based immunotherapy can eliminate tumor cells not only by blocking oncogenic signaling pathways but also by engaging immune effector cells through antibody-dependent cellular cytotoxicity (ADCC). Enhancing ADCC has therefore emerged as a crucial strategy to enhance the therapeutic efficacy of anti-cancer antibodies. This study aimed to evaluate the functional activity of a fully humanized Fc-engineered anti-CD147 monoclonal antibody, HuM6-1B9-5M, which was designed to enhance ADCC by introducing five Fc mutations (L235V/F243L/R292P/Y300L/P396L). HuM6-1B9-5M was successfully expressed in HEK293T cells and retained CD147 binding activity and specificity comparable to those of the parental antibody. Biolayer interferometry against CD147 confirmed that Fc substitutions did not adversely affect antigen-binding affinity. Functional assays using PBMC effector cells demonstrated enhanced ADCC activity of HuM6-1B9-5M relative to the wild-type antibody (HuM6-1B9-WT) against Jurkat and SupT1 cell lines, representing T-ALL and T-LBL models, respectively.

**Supplementary Information:**

The online version contains supplementary material available at 10.1038/s41598-026-52003-x.

## Introduction

T-cell acute lymphoblastic leukemia (T-ALL) and T-lymphoblastic lymphoma (T-LBL) are heterogeneous T-cell hematologic malignancies that arise from the clonal expansion of T-cell precursors and are strongly associated with poorer prognoses than their B-cell counterparts^1–3^. T-ALL/LBL are characterized by uncontrolled proliferation of immature T-lymphoblasts and their localization to different anatomical sites, mainly in the bone marrow, mediastinum, and lymphoid organs. This dissemination frequently leads to central nervous system (CNS) involvement, which represents a major site of relapse^4–6^. Despite significant advances in monoclonal antibody (mAb)-based therapies for leukemia, effective targeted treatments for T-ALL/LBL remain limited, leaving patients largely dependent on intensive, non-specific chemotherapy regimens with substantial toxicity and adverse effects^7^. Therefore, there remains a need for more effective and targeted treatment approaches for T-ALL/LBL.

The cluster of differentiation 147 (CD147), also known as extracellular matrix metalloproteinase inducer (EMMPRIN) or basigin (BSG), is a type I transmembrane glycoprotein of the immunoglobulin superfamily that is frequently upregulated in various cancers, including T cell malignancies^8,9^. CD147 plays a crucial role in regulating key oncogenic processes, such as cancer cell proliferation, invasion, metastasis, angiogenesis, glycolysis, and chemoresistance^10,11^. CD147 is also involved in the induction of matrix metalloproteinases (MMPs). MMP activation leads to extracellular matrix (ECM) degradation, thereby promoting tumor cell invasion and metastasis. Moreover, levels of MMP-2 and MMP-9 are strongly associated with leukemic cell extravasation in adult acute lymphoblastic leukemia^12^. In T-cell malignancies, CD147 is overexpressed in multiple subtypes, including T-ALL/LBL^13,14^. In T-ALL, particularly in the Jurkat T-cell line, suppressing CD147 expression through RNA interference has been shown to reduce cancer aggressiveness^15^. Collectively, these findings indicate the pathological significance of CD147 overexpression in T-cell malignancies and highlight its potential as a promising therapeutic target.

Several strategies have been developed to target CD147, including monoclonal antibodies (mAbs), antibody-drug conjugates (ADCs), and small molecule inhibitors^16–20^. Among these approaches, numerous anti-CD147 mAbs have been generated to inhibit cancer cell proliferation and induce apoptosis, either through direct blockade of CD147 signaling or via antibody internalization upon ligand binding^19,20^. For example, metuximab which targets the D1 domain of CD147, has demonstrated effective inhibition of hepatocellular carcinoma invasion and metastasis^21^. Moreover, the antibody-drug conjugate HcHAb18-DM1 exemplifies a cancer killing mechanism by undergoing internalization after binding to CD147, thereby suppressing cancer cell proliferation^22^. Beyond the mechanisms of direct blockade and internalization, the Fc region of mAbs plays a critical role in mediating immune effector functions, including antibody-dependent cellular phagocytosis (ADCP), antibody-dependent cellular cytotoxicity (ADCC), and complement-dependent cytotoxicity (CDC). These processes are mediated by macrophages, natural killer cells, and complement proteins, respectively^23^. Among these mechanisms, ADCC is a key immune effector function of therapeutic mAbs, with NK cells serving as the primary effector population. During ADCC, the Fc region of mAbs binds to Fcγ receptor IIIa (FcγRIIIa or CD16a) on NK cells, leading to NK cell activation and subsequent killing of antibody-coated cancer cells^24^.

Several clinically successful mAbs exert their antitumor activity, in part, through robust ADCC. Notable examples include the anti-human epidermal growth factor receptor 2 (HER2) mAb trastuzumab, used in breast cancer, and the anti-CD20 mAb rituximab, widely applied in the treatment of B-cell malignancies^25,26^. Consequently, extensive efforts have focused on enhancing the cytotoxic efficacy of mAbs by improving their ability to induce ADCC. One such approach involves optimizing antibody glycosylation. For example, afucosylated anti-HIV-1 broadly neutralizing antibodies (bNAbs) exhibit enhanced binding affinity to FcγRIIIa, resulting in markedly increased NK cell activation and ADCC activity in vitro, even at low antigen density^27^. In addition, Fc amino acid substitutions have been employed to enhance binding to activating FcγRIIIa while reducing binding to inhibitory FcγRIIb (CD32b), which is expressed on myeloid cells and B cells. Margetuximab, an FDA-approved anti-HER2 antibody, incorporates five amino acid substitutions (L235V/F243L/R292P/Y300L/P396L) in the Fc region that enhance affinity for FcγRIIIa, particularly in individuals with the low-affinity F/F genotype, leading to a significant enhancement of ADCC activity compared with its wild-type antibody. In addition, reduced FcγRIIb engagement has been reported for some Fc-engineered antibodies, which may further favor activating signaling in mixed effector-cell settings^28^. Other Fc-engineered mAbs, such as XmAb5574 targeting CD19 in chronic lymphocytic leukemia and MGA271 targeting B7-H3 in renal cell carcinoma and bladder carcinoma, demonstrated ADCC enhancement in vitro and in vivo, respectively^29,30^. Accordingly, these studies highlight Fc engineering as a powerful strategy to strengthen Fc-FcγRIIIa interactions and maximize ADCC-mediated tumor cell eradication, underscoring its continued development as an effective approach in antibody-based cancer immunotherapy.

In our previous studies, we characterized a fully humanized anti-CD147 mAb (HuM6-1B9) and demonstrated its ADCP activity in T-ALL as well as its ADCC activity in triple-negative breast cancers and tumor spheroids^31–33^. However, the ADCC activity of HuM6-1B9 in T-ALL/LBL has not been explored. To address this gap, we developed an Fc-optimized variant of HuM6-1B9, termed HuM6-1B9-5M (5M, denoting five Fc substitutions), by introducing five amino acid substitutions (L235V/F243L/R292P/Y300L/P396L) into its Fc region. These substitutions are identical to the Fc mutations incorporated into margetuximab. We then characterized its antigen-binding activity, binding affinity, and ADCC using the T-ALL/LBL cell lines Jurkat and SupT1. Collectively, the data suggest that the Fc-engineered mAb HuM6-1B9-5M is associated with increased ADCC activity while retaining antigen specificity and binding affinity, warranting further investigation as a potential antibody-based therapeutic for T-cell malignancies.

## Results

### Production and purification of HuM6-1B9-5M

**Fig. 1:**
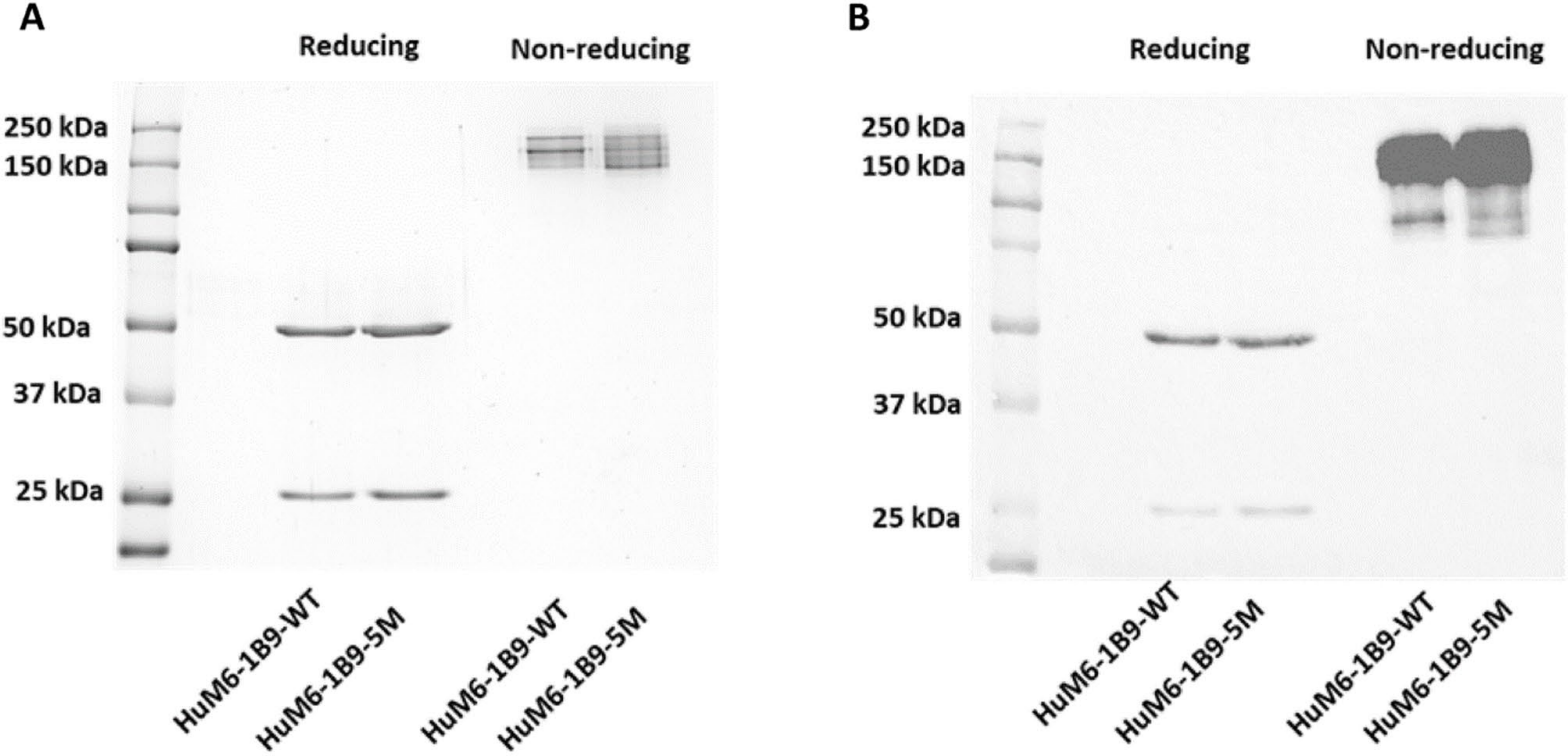
Purity and identity analysis of HuM6-1B9-5M. (**A**) The purity of HuM6-1B9-5M was determined by 10% SDS-PAGE, followed by Coomassie blue staining. (**B**) Western blot analysis was used to confirm the identity of HuM6-1B9-5M as a human IgG antibody using HRP-conjugated rabbit anti-human IgG (H + L) antibody.

For HuM6-1B9-5M production, the pVITRO1-HuM6-1B9-5M-IgG1/κ expression plasmid was transfected into HEK293T cells to establish stable antibody expression, and hygromycin B selection was applied to enrich plasmid-positive cells. The selected cells were expanded and gradually adapted to serum-free conditions using CDM4HEK293 medium, with stepwise increases from 25% to 100% serum free medium (SFM). Culture supernatants were subsequently collected, and HuM6-1B9-5M was purified by Protein G affinity chromatography. Antibody purity was examined by SDS-PAGE, demonstrating an intact IgG band migrating at ~ 150 kDa under non-reducing conditions (with minor higher-molecular-weight species), and distinct heavy and light chains at ~ 50 kDa and ~ 25 kDa under reducing conditions (Fig. 1A). Western blot analysis further confirmed the presence of human IgG, as detected by a horseradish peroxidase (HRP)-conjugated rabbit anti-human IgG (H + L) antibody (Fig. 1B). HuM6-1B9-WT was applied as a positive control antibody.

### Binding activity and affinity of HuM6-1B9-5M by indirect ELISA and biolayer interferometry (BLI)

**Fig. 2:**
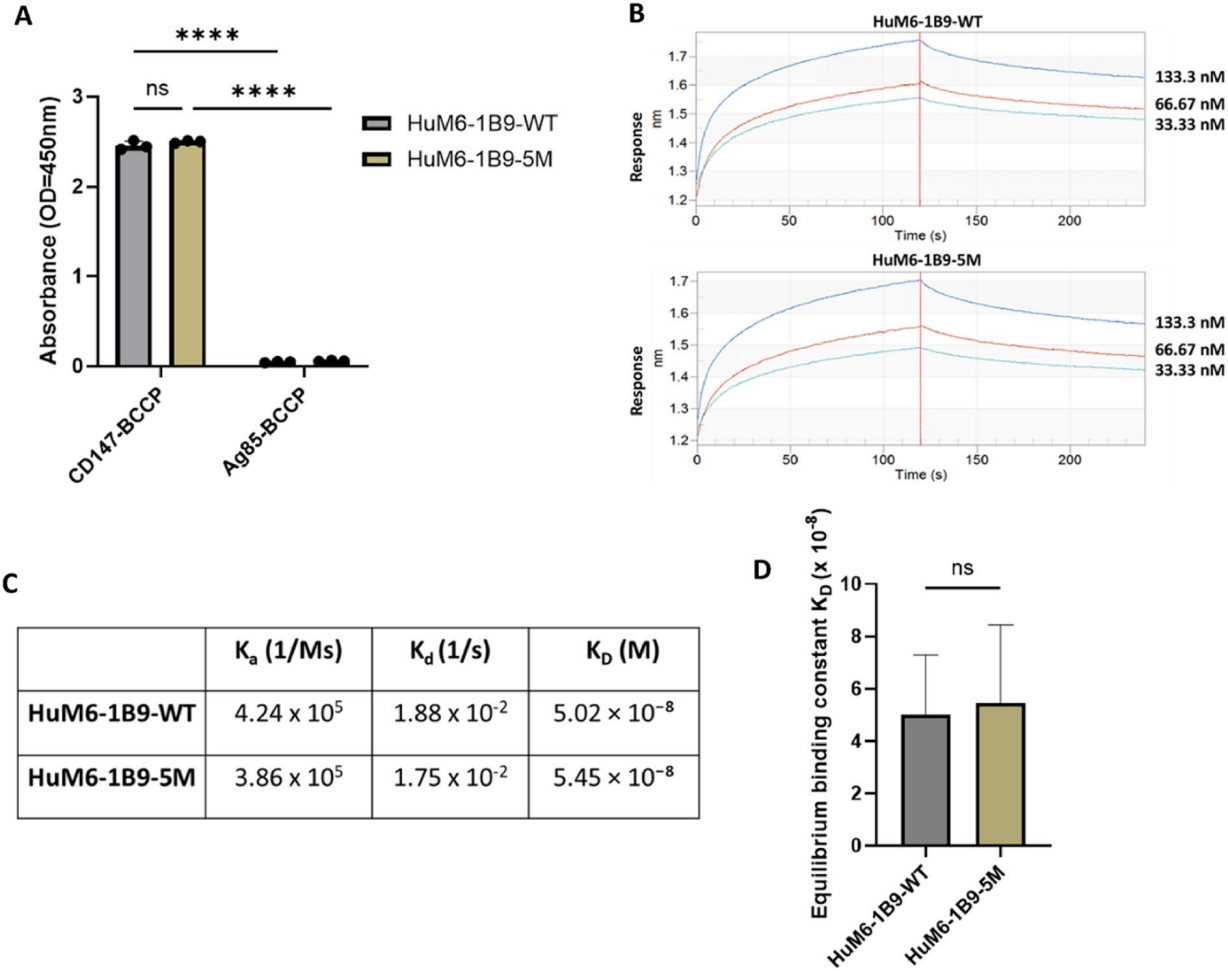
Binding activity and affinity of HuM6-1B9-5M. (**A**) Specific binding activity of HuM6-1B9-5M was measured by indirect ELISA and compared with its parental antibody, HuM6-1B9-WT, using CD147-BCCP as the target antigen and Ag85-BCCP as an irrelevant antigen control. Data represents mean ± SD from three independent experiments (*n* = 3). Statistical analysis was performed using two-way ANOVA followed by Tukey’s multiple comparisons test (NS = *P* > 0.05, ****P < 0.0001). (**B**) The BLI sensorgrams showing the binding responses of HuM6-1B9-WT and HuM6-1B9-5M towards CD147 immobilized on sensor tips at three antibody concentrations. (**C**) Table summarizing the average K_D_ values determined from three different antibody concentrations. (**D**) Comparison of K_D_ values of HuM6-1B9-WT and HuM6-1B9-5M, calculated from the ratio of dissociation (k_d_) to association (k_a_) rate constants using global fitting of the sensorgram data. Data are represented as mean ± SD and were analyzed using an unpaired t-test (NS = *P* > 0.05).

The specific binding activity of HuM6-1B9-5M to human CD147 was confirmed by indirect ELISA. HuM6-1B9-5M exhibited clear and specific binding to CD147-coated plates, with no detectable binding to the irrelevant antigen, Ag85-BCCP, while the parental antibody HuM6-1B9-WT served as a positive control (Fig. 2A). Moreover, the real-time binding kinetics were further evaluated by BLI and compared with HuM6-1B9-WT. The association and dissociation sensorgrams demonstrated comparable binding kinetics between HuM6-1B9-5M and its parental antibody (Fig. 2B). The equilibrium dissociation constants (K_D_) were 5.45 × 10⁻⁸ M for HuM6-1B9-5M and 5.02 × 10⁻⁸ M for HuM6-1B9-WT (Fig. 2C and D). These results indicated that Fc engineering did not compromise the antigen specificity or binding affinity of HuM6-1B9-5M toward CD147.

### CD147 surface expression and flow cytometric binding activity on Jurkat and SupT1 cells

**Fig. 3:**
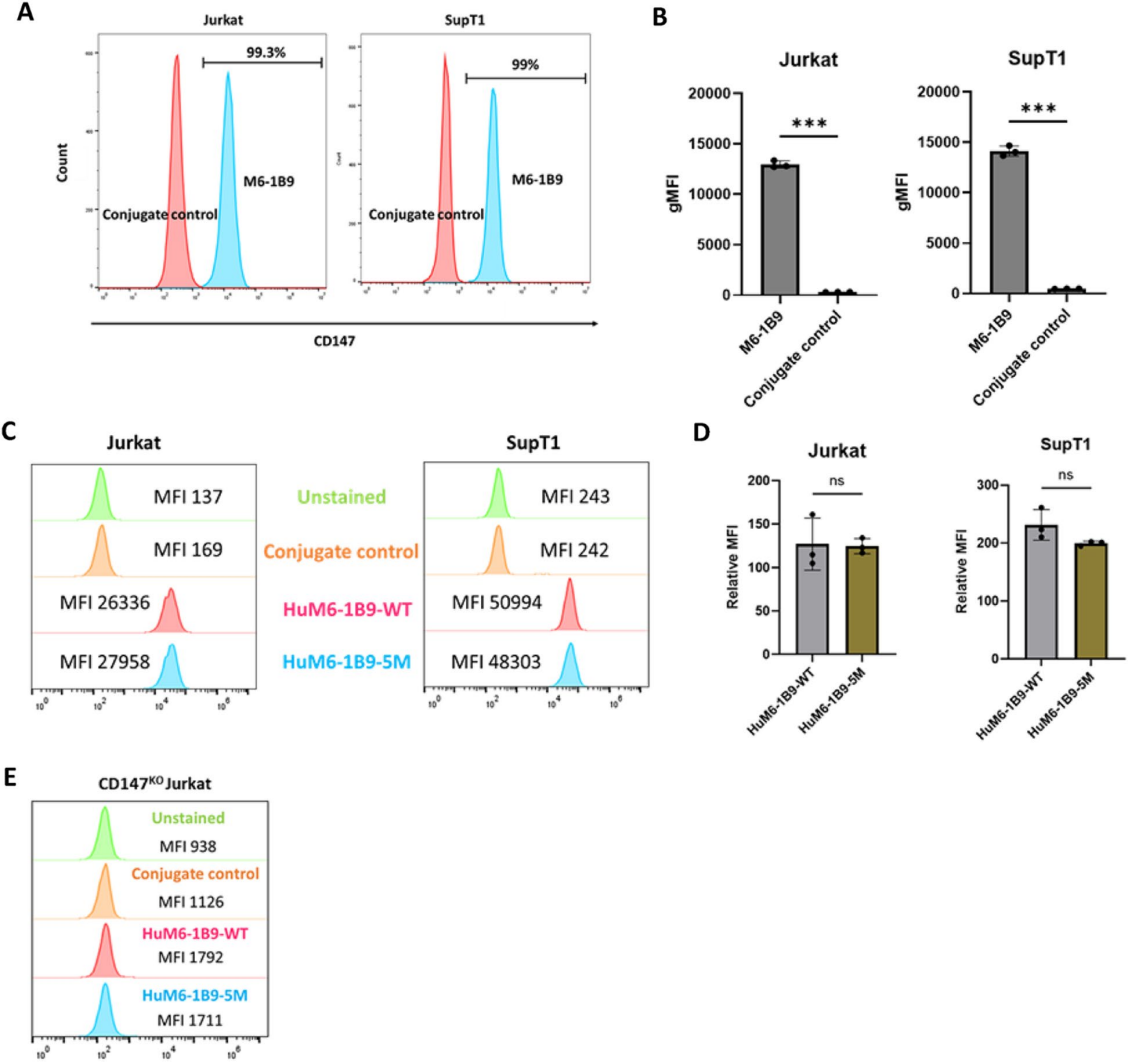
Flow cytometric analysis of CD147 surface expression and binding activity on Jurkat and SupT1 cells. (**A**) Representative histograms showing CD147 surface expression on Jurkat and SupT1 cells (blue) stained with mouse anti-CD147 mAb (M6-1B9) and detected using a FITC-conjugated F(ab′)_2_ goat anti-mouse IgG, compared with the conjugate control (pink). (**B**) Geometric mean fluorescence intensity (gMFI) of CD147 surface expression on Jurkat and SupT1 cells relative to the conjugate control. Data are presented as mean ± SD from three independent experiments and were analyzed using an unpaired t-test (***P ≤ 0.001). (**C**) Representative overlay histogram showing the binding of HuM6-1B9-5M to CD147 expressed on Jurkat and SupT1 cells. (**D**) Quantification of cell surface binding expressed as relative mean fluorescence intensity (MFI), calculated by normalizing antibody-stained MFI to the corresponding conjugate control. Data are presented as mean ± SD from three independent experiments (*n* = 3). Each dot represents one independent experiment. (**E**) Representative overlay histogram showing the binding of HuM6-1B9-5M to CD147^KO^ Jurkat cells. Statistical analysis was performed using a paired t-test (NS = *P* > 0.05).

Jurkat and SupT1 cells were harvested and stained with a mouse anti-CD147 monoclonal antibody (clone M6-1B9). A FITC-conjugated F(ab′)_2_ goat anti-mouse IgG + IgM (H + L) secondary antibody was used for detection. Flow cytometric analysis confirmed high surface expression of CD147 on both Jurkat and SupT1 cells (Fig. 3A and B), supporting their suitability as CD147-positive leukemic T cell models for subsequent functional assays. The binding activity of HuM6-1B9-5M to CD147 expressed on the surface of Jurkat and SupT1 cells was also examined by flow cytometry and compared with its wild-type counterpart, HuM6-1B9-WT. Both antibodies exhibited strong and specific surface binding, as detected by PE-conjugated secondary antibody staining. The binding of HuM6-1B9-5M was comparable to that of HuM6-1B9-WT in both cell lines, indicating that Fc engineering did not compromise cell surface antigen recognition (Fig. 3C and D). Additionally, CD147 knockout (CD147^KO^) Jurkat cells were also included as a negative control and showed no binding to either the wild-type or Fc-engineered antibodies, confirming their specificity for CD147 (Fig. 3E). Unstained cells and conjugate controls were included as negative controls.

### HuM6-1B9-5M enhances ADCC against Jurkat and SupT1 cells in PBMC co-culture

**Fig. 4:**
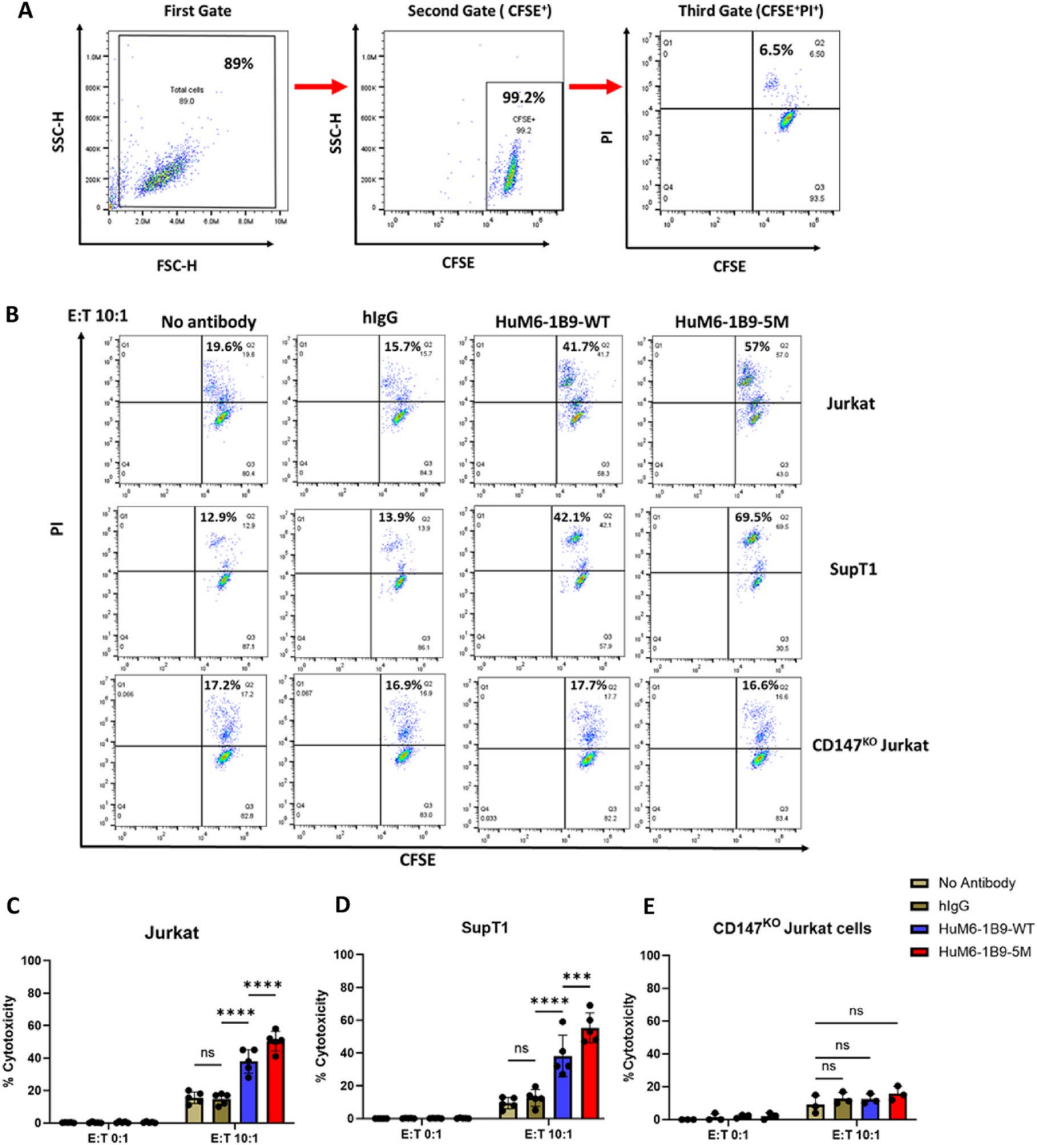
Flow cytometric analysis of HuM6-1B9-5M mediated ADCC. Jurkat and SupT1 cells were labelled with CFSE and incubated with hIgG, HuM6-1B9-WT, or HuM6-1B9-5M in the presence or absence of PBMCs. Data are shown as mean ± SD from five independent experiments using PBMCs from five different donors. (**A**) The gating strategy and analysis of ADCC-mediated target cell death were performed by flow cytometry, with dead target cells identified as double-positive cells (CFSE⁺PI⁺). (**B**) Representative flow cytometry dot plots of target cell death using PBMCs from one donor. Quantification of target cell cytotoxicity in Jurkat (**C**), SupT1 (**D**) and CD147^KO^ Jurkat (**E**) cells at E:T ratios of 0:1 and 10:1. Statistical analysis was performed using repeated-measures two-way ANOVA, with donor treated as the repeated factor, followed by Tukey’s multiple-comparison test (NS = *P* > 0.05, ***P ≤ 0.001, ****P ≤ 0.0001).

Jurkat and SupT1 cells, representing T-ALL and T-LBL, respectively, were labeled with 5,6-carboxyfluorescein diacetate succinimidyl ester (CFSE) and used as target cells in the ADCC assay. CFSE-labeled target cells were co-cultured for 4 h with 0.1 µg/mL of HuM6-1B9-WT, HuM6-1B9-5M, or hIgG as an antibody control, in the presence (effector-to-target [E:T] ratio of 10:1) or absence (E:T ratio of 0:1) of peripheral blood mononuclear cells (PBMCs). After harvesting and staining with propidium iodide (PI), target cell death was quantified as CFSE⁺PI⁺ by flow cytometry (Fig. 4A and B). Flow cytometric analysis revealed that HuM6-1B9-5M significantly enhanced target cell cytotoxicity, achieving up to ~ 60% cell death in Jurkat cells and ~ 70% in SupT1 cells at 0.1 µg/mL. In PBMC co-cultures, HuM6-1B9-5M-mediated cytotoxicity was approximately threefold higher than observed in the absence of antibody or with the hIgG control. Importantly, HuM6-1B9-5M consistently increased ADCC by an additional 10–15% points compared with its parental antibody across PBMCs from all five healthy donors (Fig. 4C and D). Moreover, the ADCC activity of HuM6-1B9-5M was evaluated against CD147^KO^ Jurkat cells and showed no enhancement, with only minimal cytotoxicity (~ 20%) observed across three independent donors (Fig. 4E), indicating that the enhanced ADCC is mediated in a CD147-specific manner. No direct cytotoxic effect was observed for either the wild-type or mutant antibodies in the absence of PBMCs, supporting that the observed cytotoxicity is effector cell-dependent and antibody-dependent under these PBMC co-culture conditions.

### HuM6-1B9-5M shows minimal off-target cytotoxicity in healthy PBMCs

**Fig. 5:**
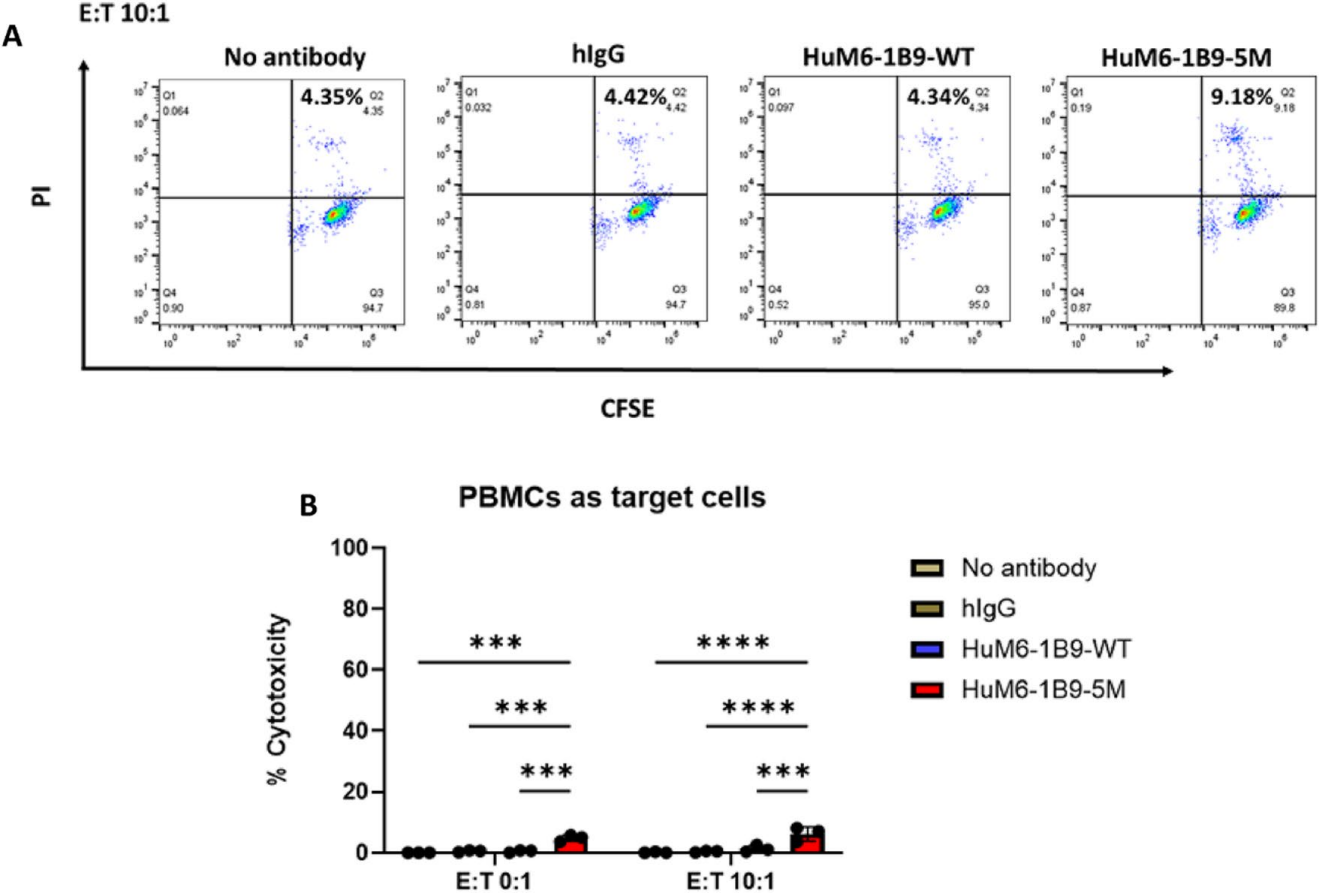
Evaluation of HuM6-1B9-5M-mediated ADCC against healthy PBMCs. PBMCs from healthy donors were used as both effector and target cells to assess the off-target cytotoxicity of HuM6-1B9-5M. (**A**) Representative flow cytometry dot plots showing target cell death using PBMCs from one healthy donor at an E:T ratio of 10:1. (**B**) Quantification of target cell cytotoxicity at E:T ratios of 0:1 and 10:1. Data are shown as mean ± SD from three independent experiments using PBMCs from three different donors. Statistical analysis was performed using repeated-measures two-way ANOVA, with donor treated as the repeated factor, followed by Tukey’s multiple-comparison test (***P ≤ 0.001, ****P ≤ 0.0001).

PBMCs from healthy donors were used as target cells to evaluate the off-target effects of the ADCC-enhanced antibody HuM6-1B9-5M. Target cells were labelled with CFSE and incubated with 0.1 µg/mL of HuM6-1B9-WT, HuM6-1B9-5M, or hIgG as an antibody control. PBMCs from three independent healthy donors were used as effector cells. After 4 h of incubation at E:T ratios of 0:1 and 10:1, target cell death was assessed as described previously. HuM6-1B9-5M induced only minimal cytotoxicity (~8%) in the presence of effector cells and ~5% in the absence of effector cells, whereas no measurable cytotoxicity was observed with the wild-type antibody or hIgG control at either E:T ratio (Figure 5).

### Enhanced ADCC mediated by HuM6-1B9-5M is dependent on FcγRIII (CD16)

**Fig. 6:**
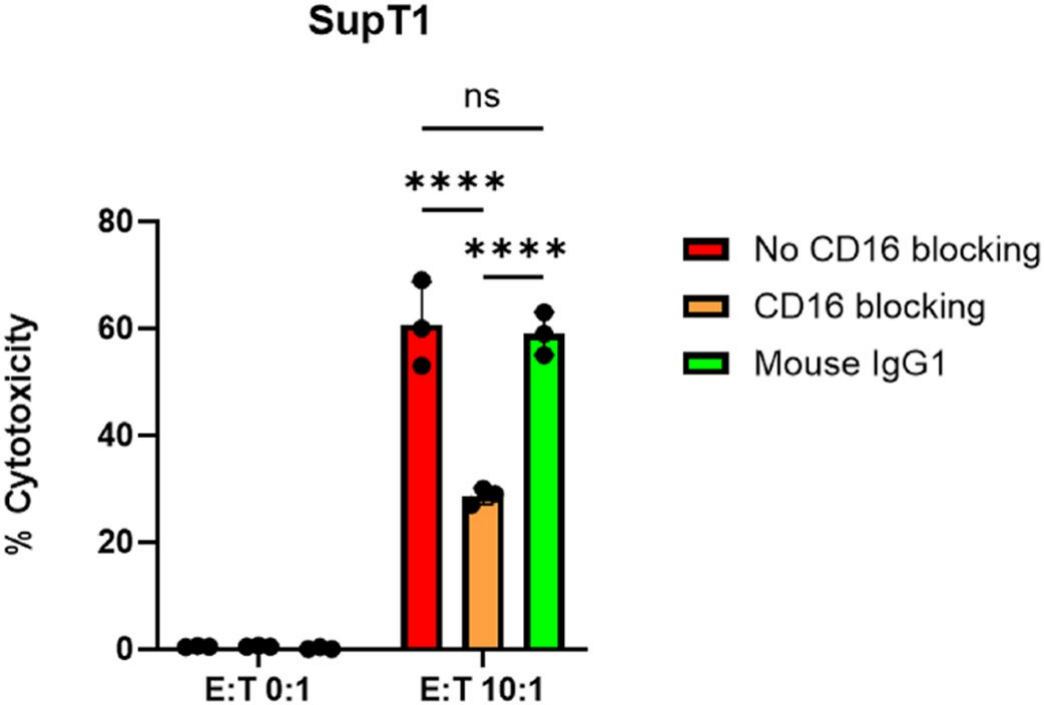
Effect of CD16 blockade on HuM6-1B9-5M-mediated ADCC. The bar graph shows the percentage of SupT1 target cell death induced by HuM6-1B9-5M in the presence or absence of anti-CD16 Ab at E:T ratios of 0:1 and 10:1. Mouse IgG1 was used as an isotype control. The data represent three independent experiments. Statistical significance was determined using repeated-measures two-way ANOVA, with donor treated as the repeated factor, followed by Tukey’s multiple-comparison test, (NS = *P* > 0.05, ****P ≤ 0.0001).

To investigate whether the enhanced ADCC activity of the Fc-optimized antibody was mediated through interaction between the Fc region and Fcγ receptor III (FcγRIII/CD16), an ADCC assay was performed using SupT1 as target cells in the presence or absence of CD16 blockade. PBMCs were preincubated with an anti-CD16 mAb or an isotype control, mouse IgG1. HuM6-1B9-5M-mediated cytotoxicity was significantly reduced by CD16 blockade compared with conditions without CD16 blocking or with isotype control across PBMCs from three independent donors (Fig. 6). In the absence of PBMCs (E:T ratio 0:1), no direct cytotoxic effect was observed. These results demonstrated that the enhanced PBMC-mediated cytotoxicity induced by HuM6-1B9-5M is dependent on Fc-FcγRIII interactions.

### HuM6-1B9-5M enhances interferon-gamma (IFN-γ) secretion by PBMCs upon co-culture with target cells

**Fig. 7:**
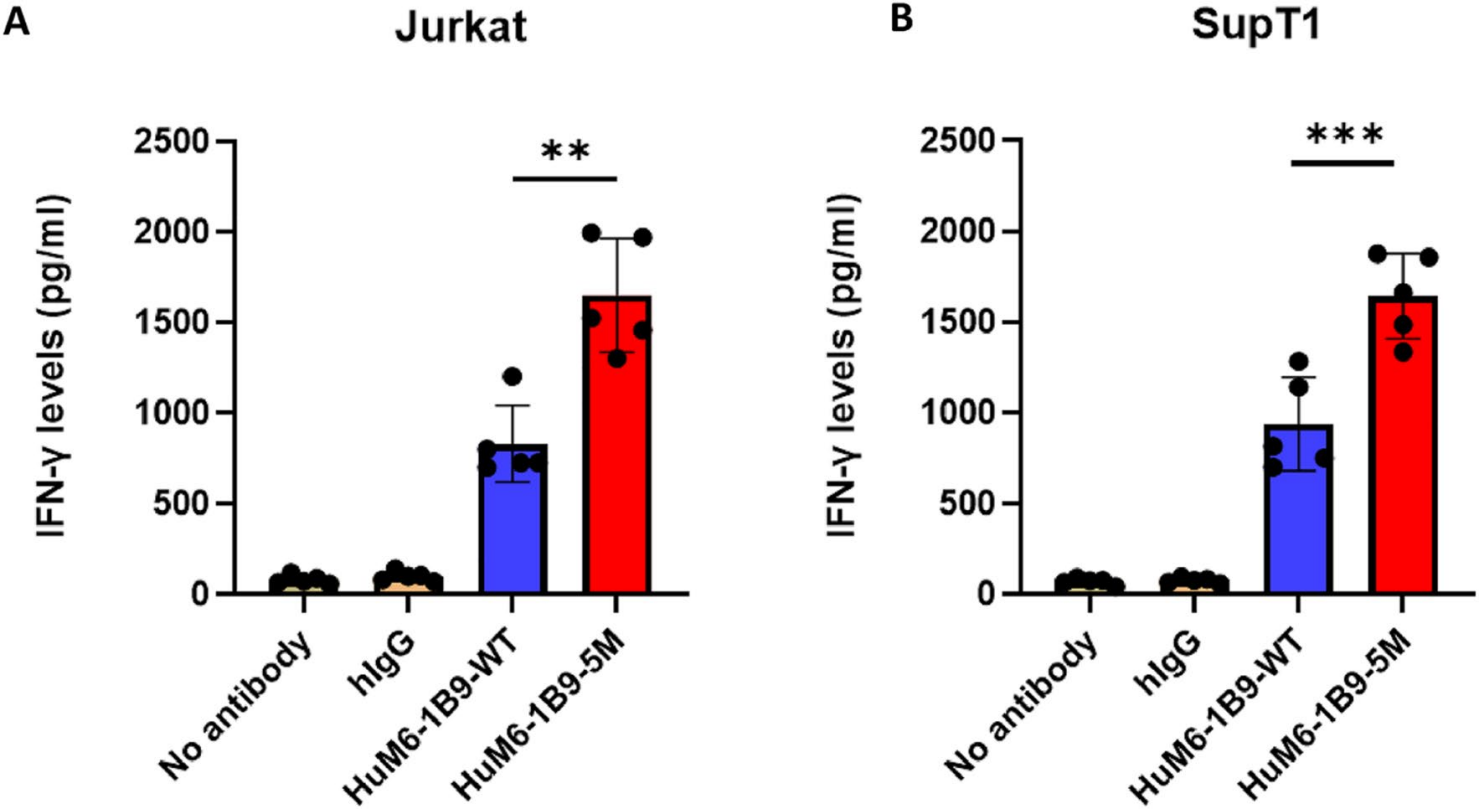
IFN-γ secretion in ADCC co-culture supernatants. IFN-γ levels were measured by ELISA in supernatants from PBMCs co-cultured with Jurkat (**A**) and SupT1 (**B**) cells at an E:T ratio of 10:1 using PBMCs from five independent donors. Each dot represents one donor (paired across conditions); bars show mean ± SD. Statistical analysis was performed using repeated-measures one-way ANOVA, with donor treated as the repeated factor. Planned pairwise comparisons between HuM6-1B9-WT and HuM6-1B9-5M were conducted using Šídák’s multiple-comparison test (**P ≤ 0.01, ***P ≤ 0.001).

To determine whether the enhanced ADCC mediated by HuM6-1B9-5M was associated with increased IFN-γ secretion, IFN-γ levels in culture supernatants from PBMCs co-cultured with Jurkat or SupT1 target cells were quantified by ELISA. Notably, significantly higher levels of IFN-γ were detected in co-cultures treated with HuM6-1B9-5M compared with those treated with HuM6-1B9-WT across all five donors. In contrast, minimal IFN-γ secretion was observed in culture supernatants treated with hIgG or without antibody (Fig. 7A and B).

## Discussion

T-ALL and T-LBL remain clinically challenging hematological malignancies due to the limited availability of effective targeted therapies^34^. Current treatment strategies rely on intensive, non-specific chemotherapy, which is associated with substantial toxicity and limited tolerability, particularly in adult and elderly patients, contributing to relapse and refractory disease^7,34–38^. Although CD147 is highly expressed on these malignancies, existing CD147-targeted approaches focus largely on direct tumor cell killing and do not fully exploit Fc-mediated immune effector mechanisms^16–18^. Importantly, the potential of Fc-engineered anti-CD147 antibodies to enhance ADCC in T-cell malignancies has not been fully investigated.

In this study, the Fc-optimized antibody HuM6-1B9-5M was successfully expressed in HEK293T cells, and its intact IgG1 structure was verified by SDS-PAGE and Western blot analyses under both reducing and non-reducing conditions, demonstrating proper assembly of heavy and light chains. Maintaining correct structure is a critical prerequisite for Fc-engineered antibodies, as improper folding or aggregation can impair Fcγ receptor binding and downstream immune effector functions such as ADCP and ADCC^39,40^. Our findings indicate that the introduced Fc substitutions (L235V/F243L/R292P/Y300L/P396L) are compatible with stable antibody production, purification, and translational development. Importantly, Fc engineering did not alter antigen recognition. HuM6-1B9-5M preserved its CD147-specific binding in indirect ELISA with absorbance (OD) values comparable to those of the parental HuM6-1B9-WT. Consistently, biolayer interferometry revealed that HuM6-1B9-5M exhibits nanomolar binding affinity (K_D_) nearly identical to that of HuM6-1B9-WT, confirming that Fc engineering did not alter binding kinetics or affinity toward CD147. These findings align with previous studies demonstrating that Fc mutations introduced to the CH2 and CH3 domains typically do not interfere with Fab-mediated antigen binding^41–43^. Importantly, retaining CD147 binding specificity is particularly crucial given the multifunctional role and broad expression of CD147 in cancer, underscoring the need for precise tumor targeting to minimize off-tumor effects.

Consistent with previous reports demonstrating elevated CD147 surface expression in T-ALL/LBL, we confirmed high CD147 surface expression on Jurkat and SupT1 cells, supporting their use as representative in vitro models for T-ALL/LBL^14,31^. CD147 overexpression has been associated with aggressive disease features and enhanced invasiveness in acute lymphoblastic leukemia, further supporting its relevance as a therapeutic target^14,15^. Flow cytometric analysis demonstrated strong binding of HuM6-1B9-5M to CD147 on both Jurkat and SupT1cells, reinforcing the translational potential of this antibody across multiple T-cell malignancy subtypes. Moreover, neither HuM6-1B9-WT nor HuM6-1B9-5M showed binding to CD147^KO^ Jurkat cells, further confirming their specificity for CD147.

In functional ADCC assays, HuM6-1B9-5M demonstrated significantly enhanced cytotoxic activity against both Jurkat and SupT1 cells when PBMCs were used as effector cells. In the absence of HuM6-1B9-WT or HuM6-1B9-5M, baseline target cell death remained at approximately 10–20%, likely reflecting NK cell-mediated cytotoxicity within the PBMC population through recognition of stress-induced ligands on tumor cells via activating receptors. In contrast, incubation with either HuM6-1B9-WT or HuM6-1B9-5M significantly enhanced tumor cell killing compared with hIgG control. Consistently, an absolute increase of approximately 10–15% points in cytotoxicity was observed with HuM6-1B9-5M compared with the wild-type antibody in all donors tested. Although modest in magnitude, such increases in ADCC are biologically meaningful. Clinical efficacy of therapeutic antibodies, including rituximab and trastuzumab, has shown that the efficacy correlates more strongly with FcγRIIIa engagement efficiency than with maximal in vitro cytotoxicity alone^23^. Notably, the enhanced ADCC mediated by HuM6-1B9-5M was observed at a low antibody concentration (0.1 µg/mL), highlighting the functional potency conferred by Fc optimization. The absence of cytotoxicity in assays lacking PBMCs supports that HuM6-1B9-5M-mediated tumor cell killing is dependent on immune effector mechanisms rather than direct induction of cell death. This phenomenon is clinically relevant, as Fc-mediated effector functions such as ADCC and ADCP are central to durable antitumor responses and the development of immune memory in antibody-based cancer therapies^44–46^. Accordingly, HuM6-1B9-5M appears to function primarily as an immunomodulatory rather than a directly cytotoxic antibody, a property that may enhance its utility in combination with other immune-based therapeutic strategies. Importantly, the enhanced ADCC activity of HuM6-1B9-5M was not observed in CD147^KO^ Jurkat cells, confirming that its cytotoxic effect is dependent on CD147 expression rather than nonspecific Fc-mediated killing. In addition, HuM6-1B9-5M induced only minimal cytotoxicity in healthy donor PBMCs used as target cells, suggesting limited off-target activity toward normal immune cells under the tested conditions. Although a low level of cytotoxicity (~ 8%) was detected, this was substantially lower than that observed in CD147-positive leukemic cell lines, supporting the preferential activity of HuM6-1B9-5M against malignant targets. Together, these findings reinforce both the target specificity and the preliminary safety profile of this Fc-engineered antibody.

Moreover, CD16 blocking experiments demonstrated that the enhanced cytotoxicity mediated by HuM6-1B9-5M is dependent on Fc-FcγRIIIa interactions. This finding is consistent with prior reports of Fc-engineered antibodies, including margetuximab, XmAb5574, and Fc-optimized anti-B7-H3 antibodies, which exploited increased FcγRIIIa binding to augment NK cell activation and target cell killing^28–30^. The reduction of ADCC upon CD16 blockade supports that the introduced Fc mutations successfully bias immune engagement toward activating, rather than inhibitory Fc receptors. In parallel, HuM6-1B9-5M significantly enhanced IFN-γ secretion from PBMCs compared with the wild-type antibody, further supporting enhanced immune activation and effector function. IFN-γ plays a pivotal role in antitumor immunity by promoting immune cell recruitment, enhancing antigen presentation, and exerting direct antiproliferative effects on malignant cells^47–49^. Increased IFN-γ levels were detected in parallel with enhanced target cell death, consistent with prior reports linking Fc-optimized antibodies to amplified cytokine release during effective ADCC responses^24,50^. These findings build upon previous work demonstrating ADCP and ADCC activity of HuM6-1B9 in T-ALL and TNBC, while extending its applicability to T-cell hematologic malignancies.

Compared with therapeutic strategies that rely on antigen internalization or antibody-drug conjugates, Fc optimization offers a modular and potentially safer approach to enhance antitumor efficacy without introducing cytotoxic payloads^51–53^. This consideration is particularly important in T-cell malignancies, where shared antigen expression between malignant and normal T cells poses a unique therapeutic challenge, as antibody-mediated targeting may result in the depletion of both cell populations and subsequent secondary T-cell immunodeficiency^4,54^. Consistent with this, our previous study demonstrated lower CD147 expression on normal lymphocytes and monocytes, and ADCC assays using normal PBMCs as target cells showed no HuM6-1B9-mediated cytotoxicity, further supporting its therapeutic potential^32^. Despite these promising results, several limitations should be acknowledged. The use of PBMCs from healthy donors may not fully recapitulate the immunosuppressive environment present in patients with T-cell malignancies, particularly in leukemia subtypes characterized by NK cell dysfunction^55,56^. Moreover, a potential limitation of CD147-targeted ADCC is the risk of on-target, off-tumor toxicity, as CD147 is not a tumor-restricted antigen. Public normal tissue datasets indicate broad CD147 expression across human tissues, highlighting the importance of careful safety evaluation prior to clinical translation^57^. At the same time, available human data on anti-CD147 antibodies do not suggest prohibitive systemic toxicity. Radiolabeled meplazumab demonstrated physiologic and tolerable biodistribution in healthy volunteers by whole-body SPECT, and a randomized phase 1 study in healthy subjects reported no serious or grade ≥ 3 treatment-emergent adverse events^58,59^. These findings support the clinical tractability of CD147 as a therapeutic target; however, they do not exclude the possibility that an ADCC-competent antibody may exhibit greater toxicity than a blocking or imaging-focused anti-CD147 antibodies. Accordingly, future studies should include direct evaluation of antibody-mediated cytotoxicity in healthy tissue subsets and other relevant normal cell types to better define the therapeutic window of CD147-directed ADCC in T-ALL. Furthermore, while in vitro ADCC assays are informative and predictive, in vivo validation using xenograft or humanized mouse models will be essential to confirm the therapeutic efficacy and safety of HuM6-1B9-5M.

In conclusion, this study demonstrates that Fc engineering of a fully humanized anti-CD147 antibody significantly enhances ADCC and IFN-γ secretion against T-ALL/LBL while preserving antigen specificity and binding affinity. These findings position HuM6-1B9-5M as a promising next-generation immunotherapeutic candidate for T-cell malignancies and provide a strong rationale for continued preclinical and translational development, including in vivo efficacy evaluation and combination immunotherapy approaches.

## Materials and methods

### Cell lines

Jurkat (clone E6-1) and SupT1 cells were purchased from American Type Culture Collection (ATCC) (Manassas, VA, USA) and cultured in Roswell Park Memorial Institute 1640 (RPMI-1640) medium supplemented with 10% heat-inactivated fetal bovine serum (FBS), 100 U/mL penicillin, 100 µg/mL streptomycin, and 2 mM L-glutamine. HEK293T cells from ATCC were cultured in Dulbecco’s Modified Eagle Medium (DMEM) (Gibco, Carlsbad, CA, USA) supplemented with 10% heat-inactivated FBS (Gibco, Carlsbad, CA, USA), 100 U/mL penicillin, and 100 µg/mL streptomycin (Gibco, Carlsbad, CA, USA). All cells were maintained at 37 °C in a 5% CO₂ incubator.

### Participants and ethical approval

Peripheral blood samples were obtained from five healthy donors for PBMC isolation. The study protocol was reviewed and approved by the Ethics Committee of the Faculty of Associated Medical Sciences, Chiang Mai University, Thailand (Approval No. AMSEC-68EX-034). Written informed consent was obtained from all participants prior to sample collection. All methods were performed in accordance with the relevant guidelines and regulations.

### Construction of the Fc-engineered HuM6-1B9-5M expression plasmid

To generate the Fc-engineered HuM6-1B9-5M construct, the VH-CH fragment containing five Fc mutations (L235V/F243L/R292P/Y300L/P396L) was commercially synthesized (GenScript, USA) and supplied pre-cloned into the pUC57 vector with flanking *Not*I and *Psi*I restriction enzyme sites. Then, the pUC57 plasmid and the parental pVITRO1-HuM6-1B9-IgG1/κ expression vector^31^ were digested with *Not*I and *Psi*I to excise the engineered VH-CH fragment and remove the native VH-CH region of HuM6-1B9, respectively. The purified Fc-engineered fragment was ligated into the digested pVITRO1-HuM6-1B9-IgG1/κ backbone, replacing the original VH-CH sequence and generating the Fc-optimized pVITRO1-HuM6-1B9-5M-IgG1/κ construct. Next, the plasmid was transformed into competent *Escherichia coli (E. coli)* XL1-blue strain. After transformation, a single colony was picked from LB agar plate containing 100 µg/mL hygromycin B for plasmid miniprep. Restriction enzyme analysis and DNA sequencing were performed to confirm correct insertion and sequence integrity of the engineered Fc region.

### Production of HuM6-1B9-5M

HEK293T cells were transfected with the pVITRO1-HuM6-1B9-5M-IgG1/κ plasmid using the TransIT-X2 Dynamic Delivery System (Mirus Bio, Wisconsin, USA) according to the manufacturer’s instructions. After the transfection, HEK293T cells were treated with 200 µg/mL hygromycin B for the selection of plasmid transfected cells. Following drug selection, cells were expanded in T75 flasks in DMEM supplemented with 10% FBS, and subsequently adapted to serum-free conditions using CDM4HEK293 (Cytiva, MXG, Wilmington, DE, USA) supplemented with 4 mM L-glutamine and 200 µg/mL hygromycin B. Adaptation to SFM was performed stepwise by sequentially increasing the proportion of SFM to 25%, 50%, 75%, and finally 100%. Cells were then cultured in 100% SFM for 4–5 days until the cell viability declined to approximately 70–80%, at which point the culture supernatant was collected. HuM6-1B9-5M was purified from the collected supernatant using a Protein G affinity chromatography column on an ÄKTA Pure protein purification system (Cytiva, MXG, Wilmington, DE, USA). Antibody concentration was determined using an ALLSHENG Nano-400 A Micro-spectrophotometer (Hangzhou Allsheng Instruments, Hangzhou, China).

### Purity assessment of HuM6-1B9-5M

The purity of HuM6-1B9-5M was assessed using 10% SDS-PAGE. Purified HuM6-1B9-5M (2 µg/lane) was mixed with either reducing or non-reducing sample buffer. Protein bands were visualized using COOMASSIE nano Protein Staining Solution (BIO-HELIX, New Taipei City, Taiwan, China). Moreover, the presence of humanized antibody was confirmed by Western blot analysis using HRP-conjugated rabbit anti-human IgG (H + L) antibody at 1:3000 dilution. Signal detection was performed using a chemiluminescent substrate and visualized by the Bio-Rad ChemiDoc Imaging System instrument (Bio-Rad, Hercules, CA, USA).

### Binding activity measurement by indirect ELISA

The specific binding activity of HuM6-1B9-5M was measured by indirect ELISA as previously described^31,32^. Briefly, 50 µL of 10 µg/mL human CD147-BCCP was coated onto 96-well plates overnight at 4 °C in a humidified chamber. All subsequent steps were performed at room temperature. After coating, wells were washed three times with washing buffer (0.05% Tween-20 in PBS) and blocked with 2% bovine serum albumin (BSA) in 0.05% Tween-20 in PBS for 1 h. After three washes, 50 µL of 1 µg/mL HuM6-1B9-5M was added to each well and incubated for 1 h. The wells were subsequently washed and incubated with HRP-conjugated rabbit anti-human IgG antibody at a 1:3000 dilution for 1 h. TMB substrate was used for color development; the reaction was then stopped with 1 N HCl, and the absorbance was measured at 450 nm using an ELISA reader (Hercuvan Lab Systems, Brixton, LDN, UK).

### Binding affinity of HuM6-1B9-5M against CD147 compared to HuM6-1B9-WT by BLI

BLI was applied to measure the real-time kinetic binding affinity of HuM6-1B9-5M in comparison to its parental antibody, HuM6-1B9-WT, using a ForteBio Octet K2 instrument (Fremont, CA, USA) as previously described^31^. Briefly, His-tagged human CD147 (Sino Biological, Beijing, China) was immobilized onto an anti-penta-HIS biosensor (HIS1K) (Sartorius, Göttingen, Germany) at a concentration of 50 µg/mL. The biosensors were equilibrated in assay buffer and then exposed to two-fold serial dilutions of HuM6-1B9-WT or HuM6-1B9-5M (33.33, 66.67, and 133.3 nM) to monitor the association phase. The dissociation phase was subsequently recorded by immersing the biosensors back into the assay buffer. All experimental steps were performed in 200 µL per well of assay buffer (0.1% BSA in 0.02% Tween-20 in PBS) at 30 °C. Data acquisition and sensorgram analysis were conducted using Octet Data Analysis 9.0 software. The fitting curves were analyzed using a 1:1 fitting mode. K_D_ values were calculated from the ratio of k_d_/k_a_.

### Flow cytometric analysis of CD147 surface expression

Jurkat and SupT1 cells were harvested and incubated with fluorescence-activated cell sorting (FACS) buffer containing 20% FBS for 30 min to block the Fc receptors. Cells were then washed three times with FACS buffer and incubated with mouse anti-CD147 mAb (clone M6-1B9) for 30 min. After three washes with FACS buffer, cells were incubated for 30 min with FITC-conjugated F(ab′)_2_ goat anti-mouse IgG + IgM (H + L) secondary antibody (ImmunoTools, Friesoythe, Germany). All steps were performed on ice to prevent antibody internalization by reducing cellular metabolic activity. Cells were then washed three times, resuspended in FACS buffer, and analyzed for CD147 surface expression using a BD Accuri C6 Plus flow cytometer (BD Biosciences, Franklin Lakes, NJ, USA). Data were processed and analyzed using FlowJo software (BD Biosciences).

### Flow cytometric analysis of binding activity of HuM6-1B9-5M

The binding activity of HuM6-1B9-5M to cell surface-expressed CD147 was further analyzed by flow cytometry. After harvesting Jurkat, SupT1 and CD147^KO^ Jurkat cells, Fc receptors were blocked by incubation with 20% FBS in FACS buffer for 30 min on ice. Cells were then stained with HuM6-1B9-5M or HuM6-1B9-WT (10 µg/mL) for 30 min on ice. Following washing, cells were incubated for 30 min on ice with a PE-conjugated goat anti-human IgM/IgG/IgA F(ab′)_2_ secondary antibody (1:250 dilution; Merck Millipore, Darmstadt, Germany). After three times washing with FACS buffer, fluorescence intensity was measured using a BD Accuri C6 Plus flow cytometer, and data were analyzed using FlowJo software.

### Assessment of ADCC mediated by HuM6-1B9-5M

PBMCs (effector cells) were isolated from heparinized peripheral blood obtained from five healthy donors using Lymphoprep solution (STEMCELL Technologies, Vancouver, Canada). Jurkat, SupT1, CD147^KO^ Jurkat cells and PBMCs, which were used as target cells, were labelled with CFSE (Sigma-Aldrich, Merck KGaA, Darmstadt, Germany) at a final concentration of 2 µM. CFSE-labelled target cells were seeded into a 96-well V-bottom plate at 2 × 10⁴ cells per well. Target cells were pre-incubated with 0.1 µg/mL HuM6-1B9-WT or HuM6-1B9-5M for 15 min at 37 °C in a 5% CO₂ incubator, and purified human IgG (hIgG) was used as an irrelevant antibody control. After pre-incubation, isolated PBMCs were added at an E:T ratio of 10:1 and incubated for 4 h at 37 °C in a 5% CO₂ incubator. Target cells alone were included as a control to determine spontaneous target cell death. Following incubation, cells were collected and stained with 5 µg/mL PI (Sigma-Aldrich, St. Louis, MO, USA) for 10 min at room temperature protected from light. Target cell death was quantified as the percentage of double-positive (CFSE⁺PI⁺) using a BD Accuri C6 Plus flow cytometer, and data analysis was performed using FlowJo software. Target cell cytotoxicity (%) was calculated as (% dead target cells – % spontaneous death) / (100 – % spontaneous death) × 100.

### CD16 blocking ADCC assay

SupT1 cells were used as target cells and were labelled with CFSE prior to incubation with 0.1 µg/mL of HuM6-1B9-5M for 15 min at 37 °C in a 5% CO₂ incubator. PBMCs were preincubated with 10 µg/mL of mouse anti-FcγRIII (CD16) antibody (clone 3G8) or mouse IgG1 isotype control (clone P3.6.2.8.1) for 30 min at room temperature. Subsequently, target cells were co-cultured with PBMCs pre-treated with mouse anti-CD16 mAb or mouse IgG1 isotype control at an E:T ratio of 0:1 and 10:1 for 4 h at 37 °C in a 5% CO₂ incubator, followed by PI staining. Target cell cytotoxicity was determined using a BD Accuri C6 Plus flow cytometer and data were processed with FlowJo software.

### Detection of IFN-γ secretion from PBMCs

Target cells, Jurkat and SupT1, were co-cultured with PBMCs at E:T ratio of 10:1 in the presence of hIgG, HuM6-1B9-WT and HuM6-1B9-5M or in the absence of antibody. The co-cultures were incubated for 4 h at 37 °C in a 5% CO_2_ incubator. Following incubation, the culture supernatants were collected, and IFN-γ levels were quantified using a Human IFN-γ ELISA Kit (FineTest, Wuhan, China).

### Statistical analysis

Statistical analyses were performed using GraphPad Prism software, version 10.1.0 (GraphPad Software, San Diego, CA, USA). Comparisons between two independent groups were conducted using unpaired and paired two-tailed Student’s t-tests. For experiments involving two independent variables without repeated measures, two-way ANOVA was performed, followed by Tukey’s multiple-comparison test. Repeated-measures two-way or one-way ANOVA was performed as appropriate, with donor treated as the repeated factor. Tukey’s multiple comparison test was used for all pairwise comparisons, whereas Šídák’s multiple comparison test was applied for planned pairwise comparisons. Statistical significance was defined as follows: NS = *P* > 0.05, *P ≤ 0.05, **P ≤ 0.01, ***P ≤ 0.001, ****P ≤ 0.0001.

## Electronic Supplementary Material

Below is the link to the electronic supplementary material.

Supplementary Material 1

## Data Availability

The DNA sequence generated during the current study is available in the GenBank repository under accession number PZ293246. Additional data supporting the findings of this study are available from the corresponding author upon reasonable request.
