# Supplementary material for: Fc engineering of a fully humanized anti-CD147 monoclonal antibody enhances ADCC against T-cell acute lymphoblastic leukemia and T-lymphoblastic lymphoma
Source: Sci Rep. 2026 May 8;16:21141. doi: 10.1038/s41598-026-52003-x (PMC13342084; doi:10.1038/s41598-026-52003-x)
Supplement: Supplementary file 1 — Supplementary Material 1 [file 41598_2026_52003_MOESM1_ESM.pdf]

# Fc Engineering of a Fully Humanized Anti-CD147 Monoclonal Antibody Enhances ADCC Against T-Cell Acute Lymphoblastic Leukemia and T-Lymphoblastic Lymphoma

Zaw Ye Htet[1,2], Thanathat Pamonsupornwichit[2], Kanokporn Sornsuwan[2,3], Natsima Viriyaadhammaa[2,5], Phatcharida Jantaree[4,5], Umpa Yasamut[2,6], Nutjeera Intasai[1,2 *], Chatchai Tayapiwatana[2,6 *]

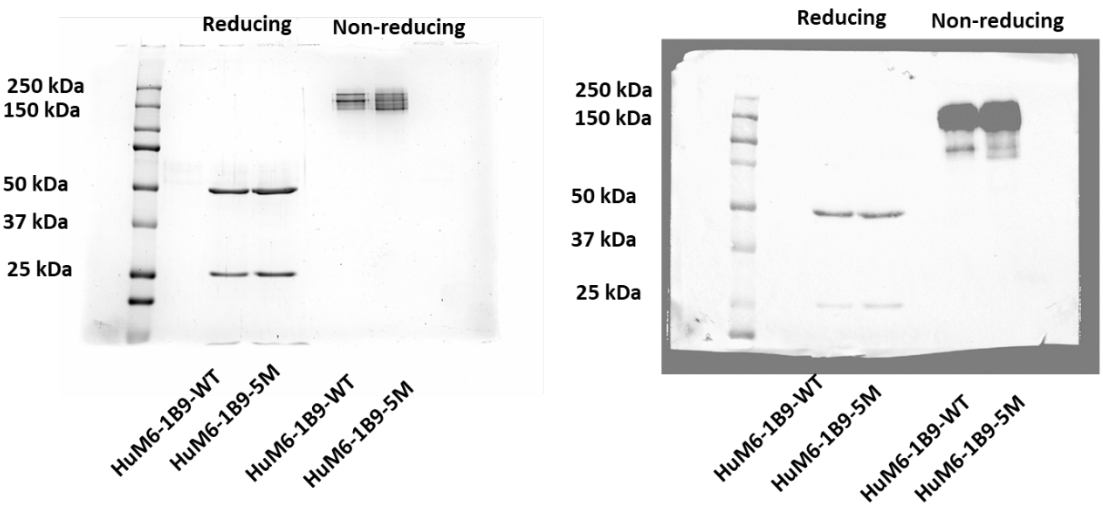


**Supplementary figure S1**. Original, uncropped gel and blot images corresponding to **Figure 1 A and B**. The cropped gel and blot panels shown in **Figure 1 A and B** were derived from the original images presented here. Full images of gel and membrane with visible edges are shown. Images included in the main figures were cropped for presentation clarity only.
